# *TULP3*: A potential biomarker in colorectal cancer?

**DOI:** 10.1371/journal.pone.0210762

**Published:** 2019-01-14

**Authors:** Ivaine Taís Sauthier Sartor, Mariana Recamonde-Mendoza, Patricia Ashton-Prolla

**Affiliations:** 1 Programa de Pós-Graduação em Genética e Biologia Molecular, Universidade Federal do Rio Grande do Sul, PPGBM-UFRGS, Porto Alegre, Rio Grande do Sul, Brazil; 2 Laboratório de Medicina Genômica, Serviço de Pesquisa Experimental, Hospital de Clínicas de Porto Alegre, LMG-SPE-HCPA, Porto Alegre, Rio Grande do Sul, Brazil; 3 Departamento de Informática Aplicada, Instituto de Informática, Universidade Federal do Rio Grande do Sul, INF-UFRGS, Porto Alegre, Rio Grande do Sul, Brazil; 4 Núcleo de Bioinformática, Serviço de Pesquisa Experimental, Hospital de Clínicas de Porto Alegre, Porto Alegre, Rio Grande do Sul, Brazil; 5 Departamento de Genética, Universidade Federal do Rio Grande do Sul, DEGEN-UFRGS, Porto Alegre, Rio Grande do Sul, Brazil; Institut National de la Recherche Scientifique, CANADA

## Abstract

Colorectal cancer (CRC) is the second most common cancer in women and the third most common cancer in men globally. The identification of differentially expressed genes associated to patient’s clinical data may represent a useful approach to find important genes in CRC carcinogenesis. Previously, the *TULP3* transcription factor was identified as a possible prognostic biomarker in pancreatic ductal adenocarcinoma. Considering that pancreatic and colorectal tissues have the same embryonic origin, we investigated the profile of *TULP3* expression in CRC hypothesizing that it may have a role in its development. We comparatively analysed *TULP3* gene expression in CRC and normal adjacent colonic tissue and assessed association of expression profiles with survival and clinicopathological information, using publicly available datasets. *TULP3* expression levels were increased in CRC when compared to the adjacent non-tumoral tissue. In addition, higher *TULP3* gene expression was associated to lymphatic and vascular invasion in colon adenocarcinoma (COAD) and rectum adenocarcinoma (READ), respectively. In summary, our results point to a possible role of *TULP3* as a diagnostic and prognostic biomarker in CRC. Additional studies are necessary to confirm these preliminary findings.

## Introduction

The global impact of cancer incidence and mortality is increasing, according to the International Agency for Research on Cancer (IARC). In 2020, more than 17 million new cancer cases will be diagnosed and about 10 million deaths in both sexes will be related to cancer [1]. Among all types of cancer, gastrointestinal (GI) tumors (those affecting the esophagus, pancreas, stomach and intestines) are responsible for approximately 20% of all diagnosed cancers worldwide and about 15% of cancer-related deaths [2].

Colorectal cancer (CRC) encompasses tumours affecting the colon and rectum. CRC is globally recognized as the third most commonly diagnosed type of cancer in men (746,000 cases, 10.0% of all cancer cases) and the second in women (614,000 cases, 9.2% of all cancer cases). Although mortality is considered low (694,000 deaths, 8.5% of total cancer deaths), less developed regions present higher mortality rates, as well as unfavorable survival for individuals diagnosed with CRC [1].

mRNA expression is recognized to play an important role in tumour cell survival and tumour progression, and comparative gene expression profiles between tumours and adjacent tissues has been important in the identification of biomarkers. [3]. Thus, in colorectal and adjacent non-tumoral tissues, analysis of differential gene expression, coupled with analysis of patient’s clinical data may represent a useful approach to identify important genes in CRC carcinogenesis [3, 4]. In a previous study based on computational analyses, Sartor *et al*. [5], identified the Tubby-like Protein 3 (*TULP3*) transcription factor as a master regulator of carcinogenesis in pancreatic ductal adenocarcinoma (PDAC) from microarray-based expression profiles. Additionally, the authors observed higher *TULP3* expression associated to a poor prognosis in PDAC patients. Given the similarity in embryonic development of pancreatic and intestinal tissues [6–10], we hypothesized that *TULP3* may also play a role in colorectal carcinogenesis.

## Methods

### Data acquisition and preprocessing

Gene expression data from patient biopsies and control samples were obtained from The Cancer Genome Atlas–TCGA Research Network (http://cancergenome.nih.gov) and Gene Expression Omnibus (GEO) [11] databases. From TCGA, we analysed the COAD and READ studies (Colon Adenocarcinoma and Rectum Adenocarcinoma, respectively) [12]. GEO datasets were downloaded under accession numbers GSE21510 [13] and GSE24514 [14]. The description of the selected datasets, including sample size, is presented in S1 Table. All data were downloaded in June 2018. Raw expression data from GSE21510 (GPL570) and GSE24514 (GPL96) studies were normalised using the RMA method implemented in the *affy Bioconductor* R package [15] see S1A and S1B Fig. Harmonized data (hg38 alignment) of RNA-Seq raw counts from the selected TCGA studies (IlluminaHiSeq) were downloaded and preprocessed using the *TCGAbiolinks Bioconductor* R package [16]. The GC-content method was used to normalise the data and quantile filtering was adopted to avoid biased gene expression values [16] (see S1C and S1D Fig). After the normalisation and filtering processes, we log-transformed the data to compare *TULP3* gene expression between tumoral and adjacent non-tumoral tissues (NT). Principal component analysis was performed in each study to filter possibly biased samples (see S2 Fig).

### Statistical analyses

Data normality assumptions were verified in all studies and the statistical tests chosen to compare *TULP3* levels were: two-tailed Mann-Whitney-Wilcoxon test (for GSE21510, COAD, and READ studies), and two tailed *t* test (for GSE24514 study).

Survival analysis was performed in COAD and READ studies using *survival Bioconductor* R package [17]. To compare the prognostic value of *TULP3* gene expression, we used the median to dichotomize the cohort. Kaplan-Meier method was used to estimate survival curves and log-rank test was used to compare the overall survival among groups. Hazard Ratios (HR) with 95% confidence intervals were calculated and *P value*s from likelihood ratio tests were considered.

To measure the association between the dichotomized *TULP3* gene expression and clinicopathological information from COAD and READ studies, we performed Pearson's Chi-squared test (with Yates' continuity correction), or Fisher exact test. All data preprocessing and analyses were performed in R 3.4.2 statistical software.

## Results

### Bioinformatics analysis of *TULP3* gene expression

**Fig 1:**
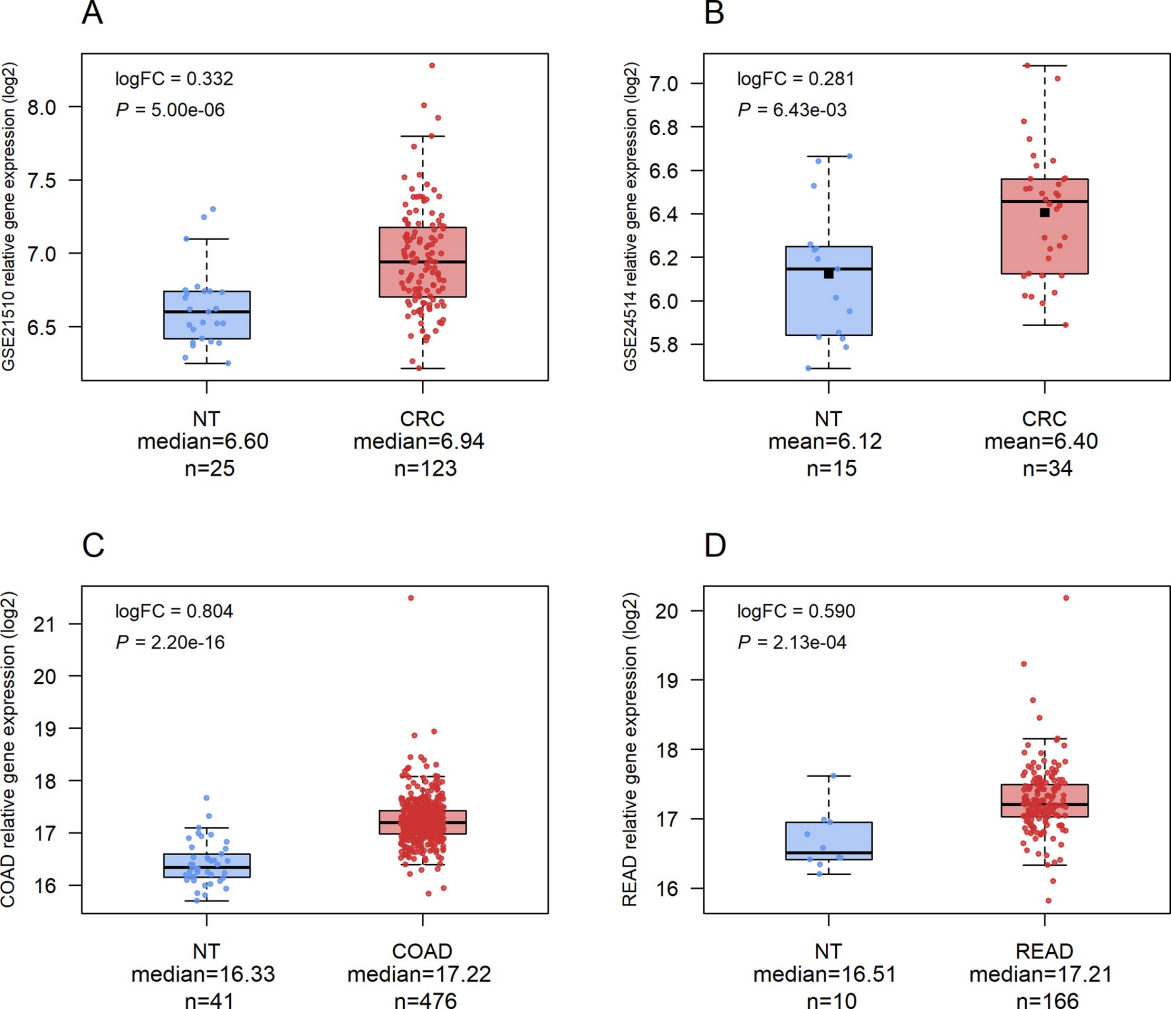
*TULP3* gene expression comparison between groups. (**A**) GSE21510, (**B**) GSE24514, (**C**) COAD-TCGA, and (**D**) READ-TCGA. (NT) Adjacent non-tumoral tissue. (CRC) Colorectal cancer. (COAD) Colon adenocarcinoma. (READ) Rectum adenocarcinoma. (logFC) Log fold-change. Median is represented as a solid black line, whereas mean is represented as a black square.

We comparatively analysed *TULP3* gene expression in GSE21510, GSE24514, COAD, and READ studies. The *TULP3* gene expression comparison between tumoral and NT samples showed significant statistical difference in all studies, with *P* = 5.00e-06, *P* = 6.43e-03, *P* = 2.20e-16 and *P* = 2.13e-04, respectively (Fig 1A–1D). In the TCGA COAD and READ studies, we observed higher fold-changes of *TULP3* gene expression in tumour samples relative to NT samples (logFC = 0.804 and logFC = 0.590, respectively). Additionally, *TULP3* expression profile from GSE21501 study was not able to differentiate primary CRC from metastatic (see S3 Fig).

### Survival analysis

In order to verify the prognostic value of *TULP3* gene expression, we performed survival analysis using the TCGA COAD and READ datasets, since this information was readily available. We used the median to dichotomize groups classified as high and low gene expression (normalised and quantile-filtered data without log-transformation). Using this approach, no survival difference was found in patients diagnosed with COAD and READ (see S4 Fig). In order to verify whether pathologic staging (pTNM) correlates to survival, we performed Kaplan-Meier curves according to pTNM classification in COAD and READ studies. We observed that there is a difference between the early stages (I and II) *versus* advanced stages (III and IV). Patients diagnosed in the early stages of the disease presented better prognosis when compared to more advanced stages (see S5 Fig). The survival analysis of staging (I, II, III and IV) of COAD and READ is depicted in S6 Fig. In READ study, the Cox proportional hazards regression analysis did not converge, therefore there is no estimates of *P* values, neither HR. Additionally, we performed survival curves based on *TULP3* gene expression and pTNM classification, to analyse the association between these variables in COAD and READ studies. To increase the sample size, we analysed early (I and II) and advanced stages (III and IV). We used the median of *TULP3* to dichotomize the groups in low and high expression. No survival difference in *TULP3* gene expression was observed in patients with early and advanced stages (see S7 and S8 Figs).

### Association of gene expression and clinical data

Chi-squared analysis was performed to verify the association among dichotomized *TULP3* gene expression and the clinical variables: gender, pathologic primary tumour extension (pT), pathologic lymph node involvement (pN), pathologic distant metastasis (pM), pathologic stage (pTNM), residual tumour, history of colon polyps, lymphatic invasion, vascular invasion, cancer status and hypermutated status. As observed in the results presented in Table 1, significant association was found in lymphatic invasion (COAD) and vascular invasion (READ), in which a higher *TULP3* expression was associated to lymphatic invasion in COAD-TCGA and to vascular invasion in READ-TCGA.

**Table 1:** Association of dichotomized *TULP3* gene expression with clinical variables of individuals diagnosed with COAD and READ.

	COAD dichotomized *TULP3* expression			READ dichotomized *TULP3* expression		
	High exp. (%)	Low exp. (%)	*P* value	High exp. (%)	Low exp. (%)	*P* value
**Gender**						
Female	119 (53.60)	103 (46.40)	0.142†	33 (44.00)	42 (56.00)	0.238†
Male	117 (46.43)	135 (53.57)		49 (54.44)	41 (45.56)	
**pT**						
T1 or T2	25 (51.02)	24 (48.98)	1.000†	12 (60.00)	8 (40.00)	1.000†
T3 or T4	72 (51.80)	67 (48.20)		29 (59.18)	20 (40.82)	
**pN**						
N0	68 (53.97)	58 (46.03)	1.000†	27 (60.00)	18 (40.00)	0.676†
N+	43 (53.75)	37 (46.25)		15 (53.27)	13 (46.73)	
**pM**						
M0	97 (56.72)	74 (43.28)	0.317†	36 (58.06)	26 (41.94)	1.000*
M1	15 (45.45)	18 (54.55)		6 (54.54)	5 (45.46)	
**pTNM**						
Stage I or II	65 (54.17)	55 (45.83)	1.000†	25 (58.14)	18 (41.86)	0.995†
Stage III or IV	46 (54.12)	39 (45.88)		16 (55.17)	13 (44.83)	
**Residual tumour**						
R0	94 (53.71)	81 (46.29)	0.614†	35 (57.38)	26 (42.62)	1.000*
R1 or R2	10 (45.45)	12 (54.55)		6 (54.54)	5 (45.46)	
**History of colon polyps**						
NO	55 (57.89)	40 (42.11)	0.371†	33 (61.11)	21 (38.89)	0.440†
YES	51 (50.50)	50 (49.50)		9 (47.37)	10 (52.63)	
**Lymphatic invasion**						
NO	**40 (44.94)**	**49 (55.06)**	**0.024†**	16 (53.33)	14 (46.67)	0.661†
YES	**63 (62.38)**	**38 (37.62)**		24 (61.54)	15 (38.46)	
**Vascular invasion**						
NO	63 (49.22)	65 (50.78)	0.195†	**21 (45.65)**	**25 (54.35)**	**0.029†**
YES	29 (61.70)	18 (38.30)		**15 (78.95)**	**4 (21.05)**	
**Cancer status**						
Tumor free	19 (48.72)	20 (51.28)	0.589†	1 (50.00)	1 (50.00)	1.000*
With tumor	92 (55.09)	75 (44.91)		41 (57.75)	30 (42.25)	
**Hypermutated status**						
NO	66 (52.80)	59 (72.20)	0.640†	37 (56.92)	28 (43.08)	1.000*
YES	19 (59.37)	13 (40.63)		2 (66.67)	1 (33.33)	
**Age mean (min-max)**	67.6 (31.2–90.0)			66.2 (31.2–90.0)		

(pT) Pathologic primary tumour extension. (pN) Pathologic lymph nodes involvement. (pM) Pathologic distant metastasis. (pTNM) Pathologic stage. Significant data are emphasized in bold.

† Pearson Chi-squared test.

* Fisher exact test.

## Discussion

TULP3 belongs to a family of proteins that possess a *tubby* domain in C-terminal, which exhibits a conserved nuclear localization and allows it to act as a transcription factor [18]. In a study of mammalian development [19], the authors have demonstrated the important role of *Tulp3* gene since its expression is ubiquitous throughout embryonic mice development and knockout of *Tulp3* leads to neural tube defects and embryonic lethality.

The Tulp3 protein negatively regulates the Hedgehog pathway in the absence of Sonic Hedgehog (Shh) ligand in mouse embryo at primary cilium (a microtubule-based membrane extension that acts as sensory signaling compartment) [20, 21]. Tulp3 is also implicated in vesicle trafficking [20, 22], since the *tubby* domain binds to PIP_2_ (phosphatidylinositol 4,5-bisphosphate), a component of cell membranes [23]. Mukhopadhyay *et al*. (2010) [22] highlighted the role of Tulp3 and intraflagellar transport complex A (IFT-A) in trafficking of a subset of G protein-coupled receptors (GPCRs) to cilia, in mouse embryo. Also, Santagata *et al*. (2001) [23] suggested that Tulp3 may play a role in GPCR signaling and gene expression regulation in Neuro-2A cell.

Several studies describe the important role of Tulp3 in embryo development however, few studies have been carried out investigating its possible role in carcinogenesis. Recently, Qian *et al*. [24] used radiogenomics analysis to identify genes associated with risk prediction in glioblastoma multiforme and *TULP3* was involved. In another study, using an *in silico* approach, increased *TULP3* gene expression was associated with poor prognosis in PDAC patients, but these results were not yet validated in clinical samples [5].

We explored the profile of *TULP3* gene expression in four independent datasets related to colorectal tissues. In all of them, we observed a statistically significant increase in *TULP3* expression levels in colorectal samples compared to NT samples, despite the small number compared to tumour samples. To classify the NT specimens, some criteria must be followed, for example, NT tissue collection should be more than 2cm away from the resected lesion margin and should not contain tumour cells in histopathological analysis [25] and, sometimes, no adjacent non-tumoral tissue is available for collection. However, even with this issue is worthy to study differences in NT and tumours samples. Recent works that used TCGA and GEO publicly available datasets also found relevant results despite the difference in sample sizes [26–28]. Taking together, these results indicate a possible role of *TULP3* in the diagnosis of CRC, however, additional studies are needed to confirm these preliminary findings. Another point to consider is that using only tumour and adjacent non-tumoral tissue we possibly added bias caused by not analysing true normal samples. Although histologically NT samples are usually considered to be normal, they could present gene expression alterations [29].

Availability of survival and clinicopathological information in the TCGA datasets allowed us to investigate the prognostic impact of *TULP3* gene expression in COAD and READ patients. Although no association of *TULP3* expression profiles and survival was identified, higher levels of *TULP3* were associated with vascular and lymphatic invasion in our study. The histological identification of lymphatic and vascular invasion has long been recognized as a potential prognostic indicator and predictor of patient outcome [30, 31]. Some studies reported that lymphatic invasion is associated with an increased risk of regional lymph node metastases [32–34] and vascular invasion with risk of distant metastases [35].

The clinicopathological characteristics, especially the tumour-node-metastasis (TNM) staging and lymphovascular invasion, are related to CRC prognostic factors. Recently, molecular markers are gaining more attention as predictive of a worse outcome, an example are the members of Wnt signaling pathway, which are known to play and important role in CRC progression [36, 37]. A molecular marker associated to tumour invasion is the β-catenin, which plays key roles in the Wnt/β-catenin signaling pathway, as well as in cellular junctions with E-cadherin. The disruption of adherens junctions is implicated in epithelial-to-mesenchymal transition (EMT) alterations, which in turn may promotes tumour spreading and, consequently, metastasis. The Wnt/β-catenin signaling pathway is also related to initiation and progression of CRC [37, 38]. A recent study, identified Tulp3 as a direct target of canonical β-catenin signaling in cerebral cortex of mice [39]. Taken together, these observations led us to hypothesize that the association found in our study between *TULP3* gene expression and, lymphatic and vascular invasion may be due to an increased activity of the β-catenin pathway. Another point to consider is that CRC is a heterogeneous and complex disease, and many studies classify both colon and rectum tumours as a common entity [40]. Evidences suggest that grouping these anatomically distinct diseases could be a clinical and biological oversimplification, therefore it would be important to study colon and rectum cancers as separated diseases, which includes differentiating them at the molecular level [40].

## Conclusions

To the best of our knowledge, this preliminary study is the first one to describe increased *TULP3* gene expression in CRC compared to adjacent non-tumoral tissue, and to associate increased gene expression to vascular and lymphatic invasion in CRC. These results point to a possible role of *TULP3* as a diagnostic and prognostic biomarker in colorectal cancer.

## Supporting information

S1 Table Description of the datasets selected to analyze *TULP3* gene expression, including the classification and sample size, and the technique employed to quantify the transcripts. (NT) Adjacent non-tumoral tissue. (CRC) Colorectal cancer. (COAD) Colon adenocarcinoma. (READ) Rectum adenocarcinoma.
(DOCX)

S1 Fig Normalisation plots. (**A**) GSE21510. (**B**) GSE24514. (**C**) COAD-TCGA. (**D**) READ-TCGA. Blue boxplots correspond to adjacent non-tumoral samples (NT) and the red ones correspond to colorectal cancer (CRC) samples.
(TIFF)

S2 Fig Principal component analysis (PCA) plots. (**A**) GSE21510. (**B**) GSE24514. (**C**) COAD-TCGA. (**D**) READ-TCGA. (PC) Principal component. (NT) Adjacent non-tumoral tissue. (CRC) Colorectal cancer. (COAD) Colon adenocarcinoma. (READ) Rectum adenocarcinoma.
(TIFF)

S3 Fig *TULP3* gene expression comparison between groups of GSE21501. *TULP3* expression profile from GSE21501 study. (NT) Adjacent non-tumoral tissue. (CRC) Colorectal cancer. Median is represented as a solid line. Equal letters above the boxplots indicate no statistical difference among the groups. We performed Kruskal-Wallis test followed by Benjamini-Hochberg correction for multiple comparisons.
(TIFF)

S4 Fig Survival probabilities in TCGA studies. We used the median to dichotomize the groups classified as high and low gene expression (normalised and quantile-filtered data without log-transformation). (**A**) Comparison of *TULP3* gene expression in Colon adenocarcinoma (COAD). (**B**) Comparison of *TULP3* gene expression in Rectum adenocarcinoma (READ). The x-axis corresponds to overall survival in months. (HR) Hazard ratio.
(TIFF)

S5 Fig Survival probabilities of early and advanced stages in TCGA studies. We grouped patients classified in stages I and II as early stage and, patients classified in stages III and IV as advanced stage. (**A**) Comparison of survival between early *versus* advanced stages in Colon adenocarcinoma (COAD). (**B**) Comparison of survival between early *versus* advanced stages in Rectum adenocarcinoma (READ). The x-axis corresponds to overall survival in months. (HR) Hazard ratio.
(RAR)

S6 Fig Survival probabilities of pathologic staging (pTNM) in TCGA studies. (**A**) Comparison of survival among pNTM stages in Colon adenocarcinoma (COAD). (**B**) Comparison of survival among pNTM stages in Rectum adenocarcinoma (READ). The x-axis corresponds to overall survival in months. (HR) Hazard ratio. In READ study the Cox proportional hazards regression analysis did not converged, therefore there is no estimates of *P* values neither HR.
(RAR)

S7 Fig Survival probabilities of *TULP3* gene expression in early and advanced stages in COAD-TCGA study. We used the median of *TULP3* to dichotomize the groups in low and high expression for early and advanced stages. (**A**) Comparison of survival between early stages (I and II). (**B**) Comparison of survival between advanced stages (III and IV). The x-axis corresponds to overall survival in months. (HR) Hazard ratio.
(RAR)

S8 Fig Survival probabilities of *TULP3* gene expression in early and advanced stages in READ-TCGA study. We used the median of *TULP3* to dichotomize the groups in low and high expression for early and advanced stages. (**A**) Comparison of survival between early stages (I and II). (**B**) Comparison of survival between advanced stages (III and IV). The x-axis corresponds to overall survival in months. (HR) Hazard ratio.
(RAR)
