# Supplementary material for: TULP3: A potential biomarker in colorectal cancer?
Source: PLoS One. 2019 Jan 14;14(1):e0210762. doi: 10.1371/journal.pone.0210762 (PMC6331117; doi:10.1371/journal.pone.0210762)
Supplement: S1 Table — (NT) Adjacent non-tumoral tissue. (CRC) Colorectal cancer. (COAD) Colon adenocarcinoma. (READ) Rectum adenocarcinoma. (DOCX) [file pone.0210762.s001.docx]

| Selected datasets | Classification of samples (n) | Technique used to  quantify transcripts (platform) |
| --- | --- | --- |
| GSE21510 | NT (25) | Microarray  Affymetrix Human Genome  U133 Plus 2 (GPL570) |
|  | CRC (123) |  |
| GSE24514 | NT (15) | Microarray  Affymetrix Human Genome  U133 A (GPL96) |
|  | CRC (34) |  |
| COAD-TCGA | NT (41) | Harmonized data  (hg38 alignment)  RNA-Seq  Illumina HiSeq |
|  | COAD (476) |  |
| READ-TCGA | NT (10) | Harmonized data  (hg38 alignment)  RNA-Seq  Illumina HiSeq |
|  | READ (166) |  |
