# Supplementary figures and images for: TULP3: A potential biomarker in colorectal cancer?
Source: PLoS One. 2019 Jan 14;14(1):e0210762. doi: 10.1371/journal.pone.0210762 (PMC6331117; doi:10.1371/journal.pone.0210762)

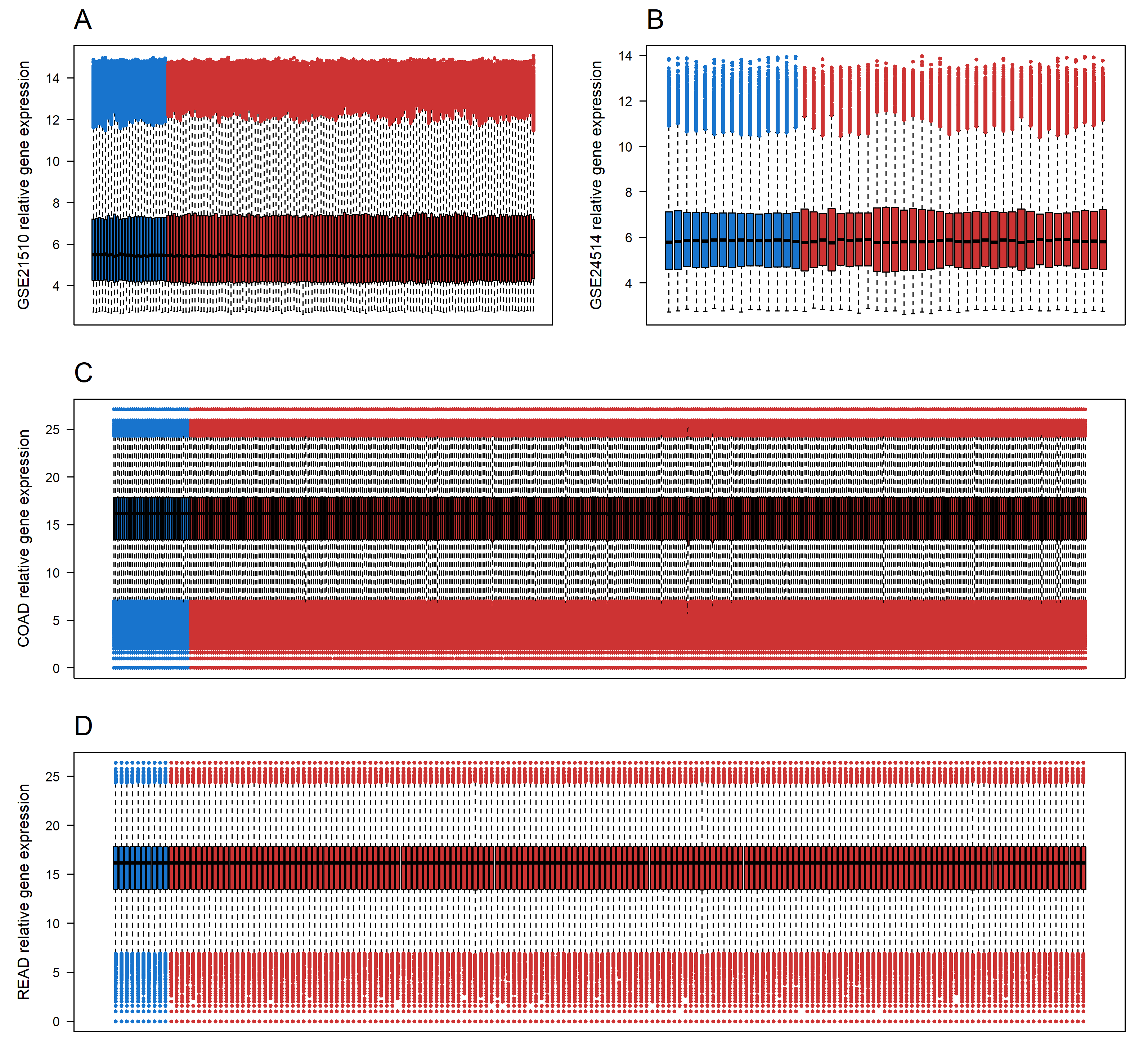

Supplement: S1 Fig — (A) GSE21510. (B) GSE24514. (C) COAD-TCGA. (D) READ-TCGA. Blue boxplots correspond to adjacent non-tumoral samples (NT) and the red ones correspond to colorectal cancer (CRC) samples. (TIFF) [file pone.0210762.s002.tiff]

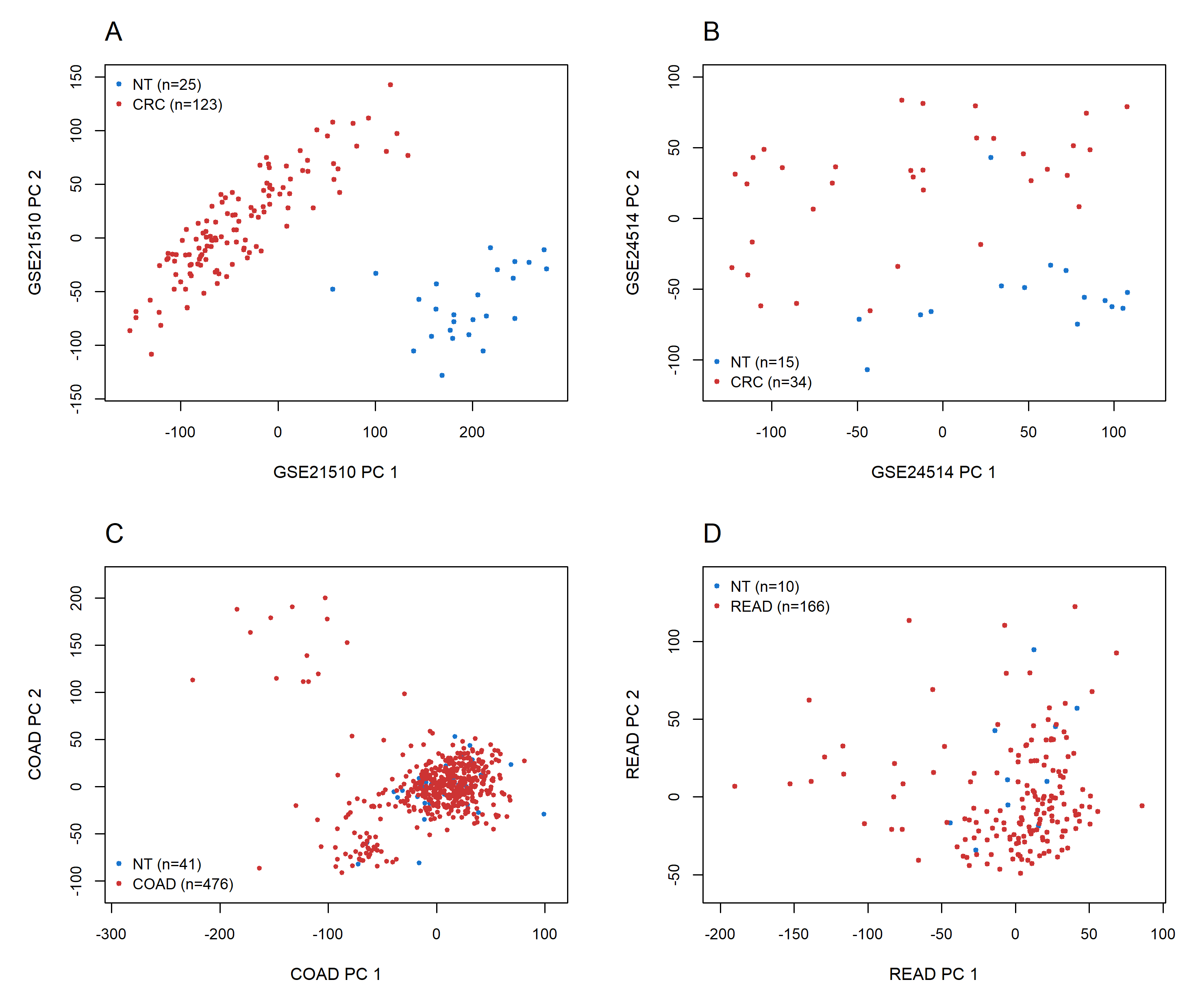

Supplement: S2 Fig — (A) GSE21510. (B) GSE24514. (C) COAD-TCGA. (D) READ-TCGA. (PC) Principal component. (NT) Adjacent non-tumoral tissue. (CRC) Colorectal cancer. (COAD) Colon adenocarcinoma. (READ) Rectum adenocarcinoma. (TIFF) [file pone.0210762.s003.tiff]

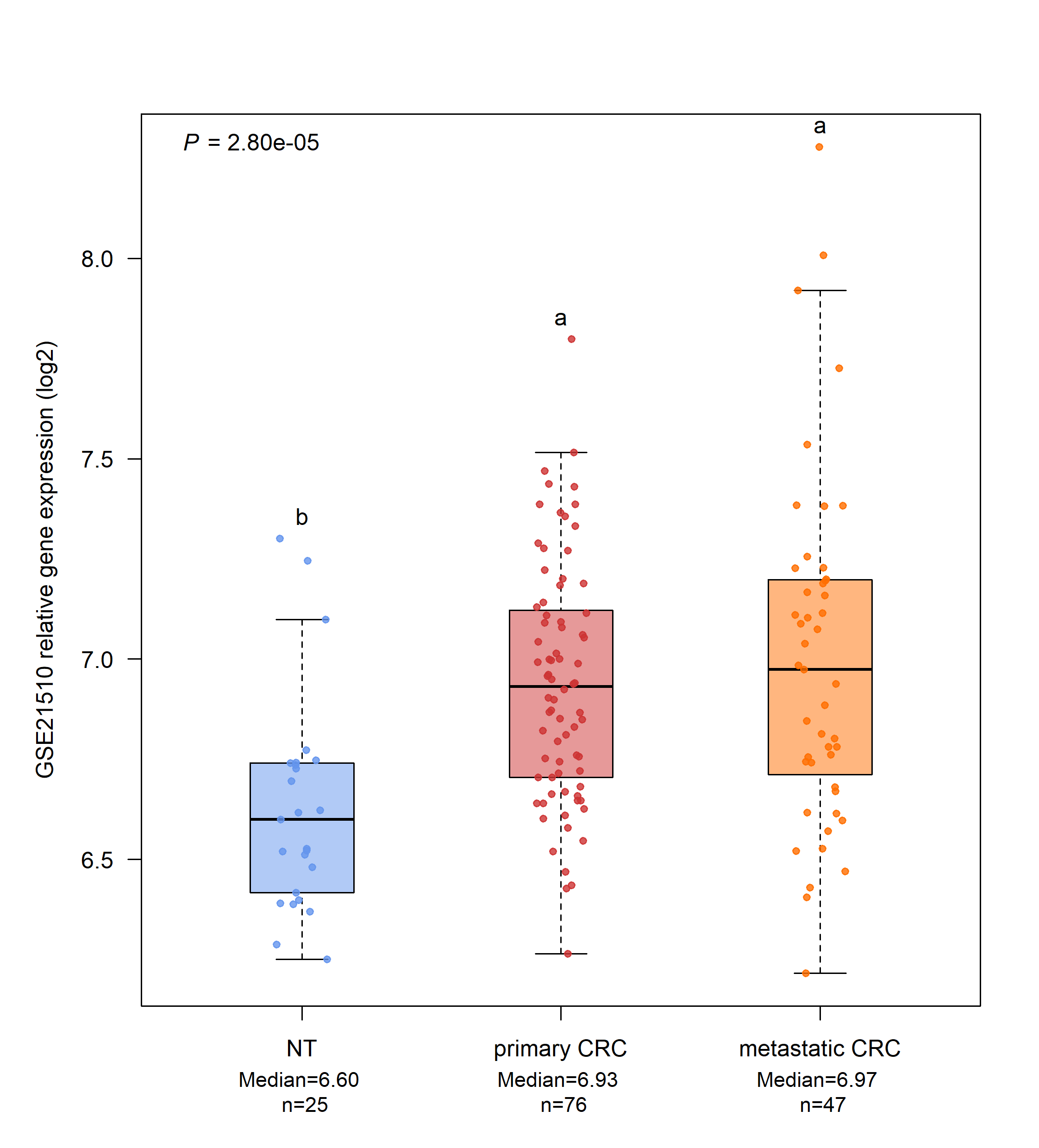

Supplement: S3 Fig — TULP3 expression profile from GSE21501 study. (NT) Adjacent non-tumoral tissue. (CRC) Colorectal cancer. Median is represented as a solid line. Equal letters above the boxplots indicate no statistical difference among the groups. We performed Kruskal-Wallis test followed by Benjamini-Hochberg correction for multiple comparisons. (TIFF) [file pone.0210762.s004.tiff]

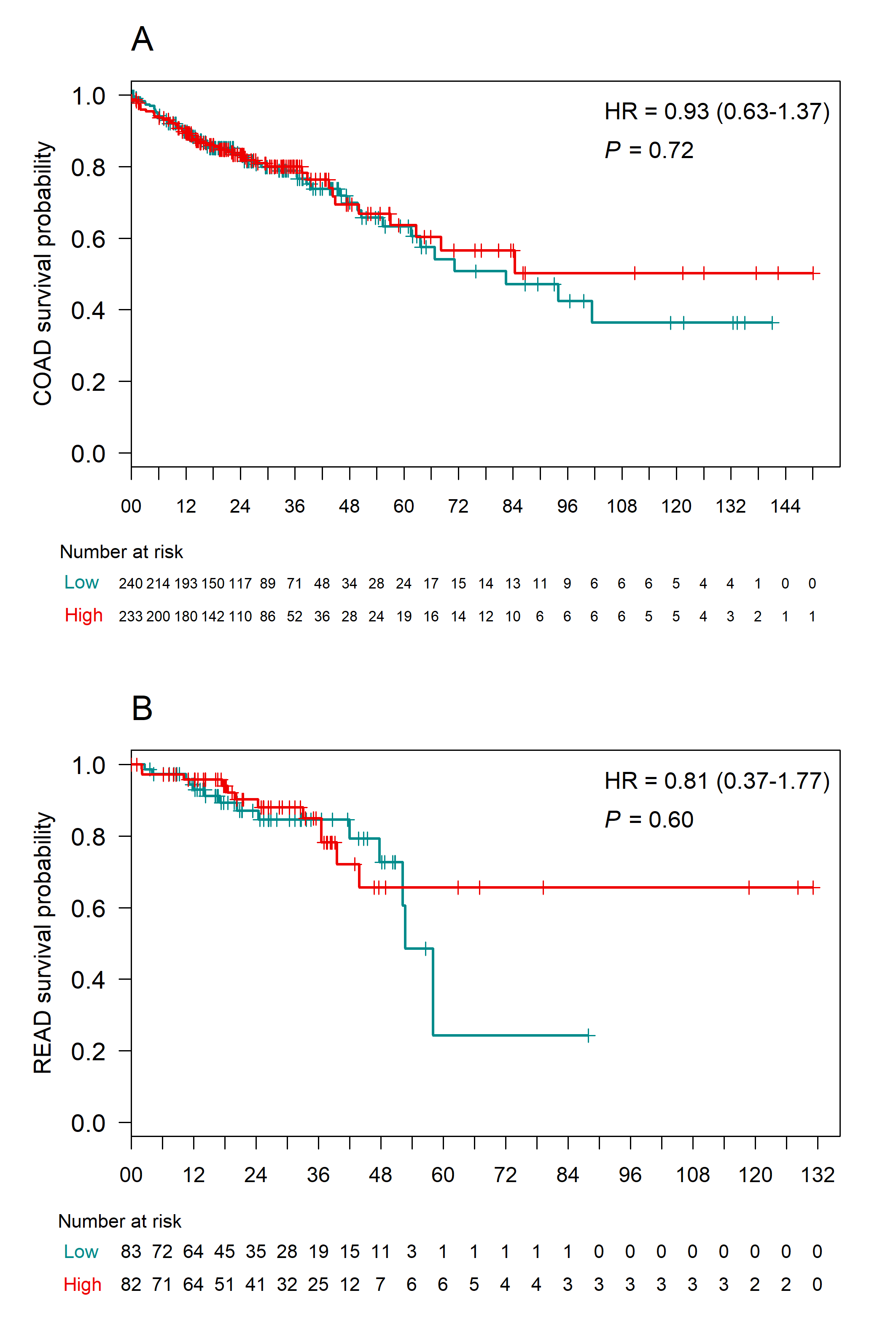

Supplement: S4 Fig — We used the median to dichotomize the groups classified as high and low gene expression (normalised and quantile-filtered data without log-transformation). (A) Comparison of TULP3 gene expression in Colon adenocarcinoma (COAD). (B) Comparison of TULP3 gene expression in Rectum adenocarcinoma (READ). The x-axis corresponds to overall survival in months. (HR) Hazard ratio. (TIFF) [file pone.0210762.s005.tiff]
